# The Potential Role of Bioactive Plasmalogens in Lung Surfactant

**DOI:** 10.3389/fcell.2021.618102

**Published:** 2021-02-16

**Authors:** Ruijiang Zhuo, Pu Rong, Jieli Wang, Rokshana Parvin, Yuru Deng

**Affiliations:** ^1^Eye Hospital, School of Ophthalmology and Optometry, School of Biomedical Engineering, Wenzhou Medical University, Wenzhou, China; ^2^Wenzhou Institute, University of Chinese Academy of Sciences, Wenzhou, China

**Keywords:** lung surfactant, plasmalogen, tubular myelin, lamellar bodies, antioxidant, cubic membrane

## Abstract

Neonatal respiratory distress syndrome (NRDS) is a type of newborn disorder caused by the deficiency or late appearance of lung surfactant, a mixture of lipids and proteins. Studies have shown that lung surfactant replacement therapy could effectively reduce the morbidity and mortality of NRDS, and the therapeutic effect of animal-derived surfactant preparation, although with its limitations, performs much better than that of protein-free synthetic ones. Plasmalogens are a type of ether phospholipids present in multiple human tissues, including lung and lung surfactant. Plasmalogens are known to promote and stabilize non-lamellar hexagonal phase structure in addition to their significant antioxidant property. Nevertheless, they are nearly ignored and underappreciated in the lung surfactant-related research. This report will focus on plasmalogens, a minor yet potentially vital component of lung surfactant, and also discuss their biophysical properties and functions as anti-oxidation, structural modification, and surface tension reduction at the alveolar surface. At the end, we boldly propose a novel synthetic protein-free lung surfactant preparation with plasmalogen modification as an alternative strategy for surfactant replacement therapy.

## Introduction

In 1959, Avery and Mead reported that the saline extracts of lungs from premature infants succumbing to neonatal respiratory distress syndrome (NRDS) were surfactant deficient as compared with those newborn dying of other causes (Halliday, 2017). This discovery spurred enormous interest in the following lung surfactant research. Decades later, pulmonary surfactant replacement therapy was proved to effectively reduce the morbidity and mortality of NRDS (Kim and Won, 2018). According to clinical studies, animal-derived surfactants are the more desirable choice than currently available protein-free synthetic ones (Ardell et al., 2015; Kim and Won, 2018). Nevertheless, the synthetic surfactant preparations produced in the laboratory have significant advantages in purity, reproducibility, safety, and quality control as compared with the animal-derived products (Notter et al., 2007). Although two major surfactant membrane proteins, SP-B and SP-C, are considered to be critical for the adsorption and spreading of surfactant film at the air–water interface (Johansson and Curstedt, 1997), it is still difficult to prepare artificial surfactant with these two surfactant proteins (Walther et al., 2019). SP-B molecule is too big and structurally complex to be synthesized by organic chemistry methods; also the expression of functionally active recombinant SP-B has not been successful yet (Curstedt et al., 2013; Parra and Pérez-Gil, 2015). SP-C is also difficult to obtain due to its extreme hydrophobicity and structural instability, especially in its pure form (Curstedt et al., 2013; Parra and Pérez-Gil, 2015).

Although the synthetic surfactant preparations can be produced in the presence of synthetic peptides that are simplified surrogates of the surfactant proteins, they are still not available in the market (Hentschel et al., 2020). The increased understanding of mechanisms involved in the formation and preservation of surfactant film at alveolar surface has led to uncover a bioactive phospholipid (PL) component in lung surfactant, plasmalogen, which has been somehow underappreciated in lung surfactant research; and its unique features as structural attribute and antioxidant allow us to boldly propose new protein-free artificial surfactant preparations with plasmalogen modification as an alternative strategy for effective surfactant replacement therapies.

## Lung Surfactant: Composition, Structure, and Function

The respiratory surface of the mammalian lung is stabilized by pulmonary surfactant, a membrane-based system. The primary function of lung surfactant is to minimize the surface tension at alveolar air–liquid interface, optimize the mechanics of breathing, and avoid alveolar collapse, especially at the end of expiration (Parra and Pérez-Gil, 2015). Lung surfactant is synthesized, stored, secreted, and recycled through type II alveolar cells (Notter et al., 2007), which emerge at the 24th gestation week and mature at the 34th–36th gestation week. The preterm babies (<34 weeks) with insufficient lung surfactant have much higher risk of developing NRDS, the main cause of perinatal mortality.

**FIGURE 1:**
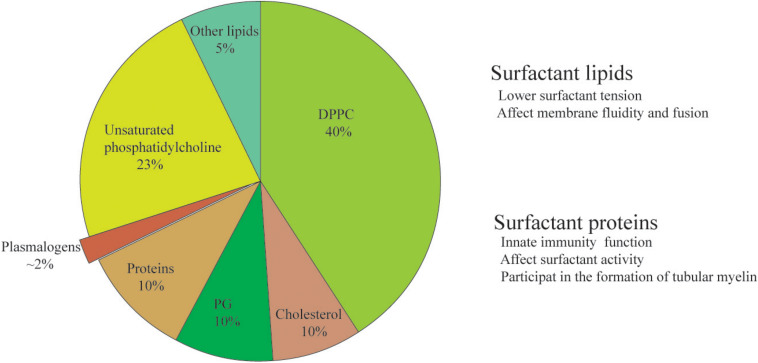
The composition and function of lung surfactant. The constitution of phospholipids% might be slightly different in different publications, and the discrepancy might be due to differences in the source (animal species) and the method of extraction (either from lavage or from whole minced tissue). Plasmalogen, a minor yet bioactive ether phospholipid of lung surfactant, is the ethanolamine and choline phospholipids vs. its ester counterpart.

The composition and biosynthesis of lung surfactant are only better understood since 1950s due to the start of recognition of NRDS (Notter, 2000). Lung surfactant is a mixture of lipids (90%) and proteins (10%) (Figure 1). In mammals, dipalmitoylphosphatidylcholine (DPPC) contributes the largest fraction of total lung surfactant. DPPC, a di-saturated PL is considered to be indispensable to allow for a reduction of surface tension at alveolar air–water interface (Wüstneck et al., 2005; Olmeda et al., 2017). Phosphatidylglycerol (PG), the second most abundant PL in lung surfactant mixture (Pfleger et al., 1972; Hallman and Gluck, 1975), may improve the property of surfactant in stabilizing the alveoli (Hallman et al., 1977; Orgeig et al., 2003). The rest of the lipid fraction is constituted by other polyunsaturated PLs including bioactive plasmalogen and cholesterol as well.

The content of plasmalogens in alveolar surfactant has been rarely investigated. The concentration of total plasmalogens is around 2% in rat surfactant (Rüstow et al., 1994) and 5–6% in dog surfactant (Rana et al., 1993). There is about 1.2% plasmalogens extracted from total lipids of pig surfactant (Body, 1971). The relative percentage of plasmalogens in the term healthy infants is 1.8 ± 0.9% (Rüdiger et al., 2000). Plasmalogen phosphocholine (PC) was first identified in the mammalian lung surfactant preparations obtained from both adult cow and lamb fetal pulmonary lavage (Rana et al., 1993). Of particular interest, the amount of plasmalogen PC in these preparations is reported to be unexpectedly comparable with that of PG, the second most abundant PL in lung surfactant lipid mixture at the fractional level (Rana et al., 1993). Thus, the presence of plasmalogen PC in pulmonary surfactant might have significant impact on animal and human physiology and suggest new directions of biochemical and biophysical studies of lung surfactant (Rana et al., 1993). Another minor yet important component of lung surfactant lipids is cholesterol, which may optimize the surfactant activity (Orgeig et al., 2003) and is considered crucial as well in surfactant function.

**FIGURE 2:**
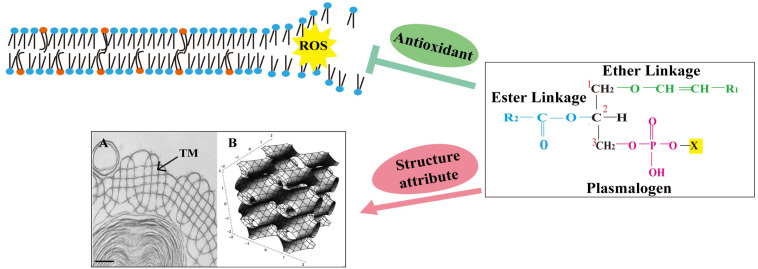
The chemical structure of plasmalogen and its potential role in lung surfactant. Plasmalogens are a class of phospholipids carrying a vinyl ether bond at sn-1 and an ester bond at sn-2 position of the glycerol backbone. The vinyl ether bond at sn-1 position is highly prone to attack by reactive oxygen species (ROS). This was proposed to prevent the oxidative damage of polyunsaturated fatty acids and other vulnerable membrane lipids, suggesting a role for plasmalogens as sacrificial molecules (antioxidant). Plasmalogens also promote the formation of non-lamellar membrane structures, including tubular myelin (TM) (a deformed P-cubic structure). Orange spheres mark plasmalogens, and blue spheres mark other phospholipids. **(A)** Transmission electron microscopy (TEM) image containing TM structure (arrow) was adapted from Williams (1977) with permission; scale bar = 100 nm. **(B)** A tP model of TM bilayer structure with the minimal surface describes the center of the bilayer. Adapted from Larsson and Larsson (2014) with permission.

As for the protein part, three out of total four surfactant proteins present in the alveolar hypophase (SP-A, SP-B, and SP-C) are membrane associated, and even with small amounts, they may have profound effect on surfactant membrane structure and function (Casals, 2001; Serrano and Pérez-Gil, 2006; Cabré et al., 2018). SP-A is able to bind to a wide variety of microorganisms (Lawson and Reid, 2000; Ariki et al., 2012), although it is hydrophilic, and together with SP-B (Suzuki et al., 1989; Knudsen and Ochs, 2018), it may participate in the formation of tubular myelin (TM), a liquid crystal membrane structure found within the alveolar space (Figure 2A). The structure of TM and its ability of being viscoelastically deformed have been suggested to explain the mechanism behind the reduction of work required for air breathing (Ninham et al., 2017). The addition of Ca^2+^ also promoted lamellar to hexagonal (H_II_) transitions in the mixtures of phosphatidylethanolamine (PE) and phosphatidylserine (Cullis et al., 1978). SP-B and SP-C, the hydrophobic surfactant proteins, have been demonstrated crucial for surfactant activity inside the alveoli, and their incorporation into the lipid mixtures could facilitate proper interfacial adsorption, film stability, and re-spreading abilities of lung surfactant (Wang et al., 1996; Cruz et al., 2000; Serrano and Pérez-Gil, 2006). Meanwhile, both SP-A and SP-D are part of the innate immune system at alveolar surface and may regulate the functions of other important innate immune cells such as macrophages (Vaandrager and van Golde, 2000; Matalon and Wright, 2004; Pastva et al., 2007; Kharlamova et al., 2020).

## The Significance of Plasmalogens

Plasmalogens are a class of PLs carrying a vinyl ether bond at sn-1 and an ester bond at sn-2 position of the glycerol backbone (Nagan and Zoeller, 2001; Wallner and Schmitz, 2011; Jiménez-Rojo and Riezman, 2019). They constitute 15–20% of total PLs of cell membranes (Braverman and Moser, 2012). Plasmalogens are abundant in the brain, retina, leukocytes (immune cells), sperm, heart, and skeletal muscle in the mammals (Braverman and Moser, 2012). They are also concentrated in specialized membranes, such as myelin, and secreted membranes such as synaptic vesicles and lung surfactant (Braverman and Moser, 2012). The physiological role of plasmalogens in cells has been proposed to range from free radical scavenging to promoting membrane fusion; it has been still difficult to assess their biophysical roles in cell membrane partly due to their variable concentration in different cell types and the change during development (Koivuniemi, 2017).

Plasmalogens as endogenous antioxidants to protect cell membrane PLs and lipoprotein particles against oxidative damage are still controversial (Yavin and Gatt, 1972; Khaselev and Murphy, 1999; Farooqui and Horrocks, 2001; Zemski Berry and Murphy, 2005; Richard et al., 2008). However, the high susceptibility of vinyl ether bond of plasmalogens to reactive oxygen species (ROS) and traces of acids has suggested their sacrificing role as a first-line defense system in a biological system (Yavin and Gatt, 1972; Khaselev and Murphy, 1999; Farooqui and Horrocks, 2001; Zemski Berry and Murphy, 2005; Richard et al., 2008). Plasmalogen-deficient cultured cells and animals have long been known to be more sensitive to oxidative damage than their wild-type counterparts, strongly suggesting that plasmalogens may act as an endogenous antioxidant in cells (Reiss et al., 1997; Morand et al., 1988; Zoeller et al., 1988; Nagan and Zoeller, 2001; Lessig and Fuchs, 2009; Wallner and Schmitz, 2011; Luoma et al., 2015). Cellular plasmalogens are also reported to act as antioxidants against ultraviolet light-induced lipid peroxidation (Zoeller et al., 1988). Moreover, plasmalogens seem to be able to act as antioxidants to protect low-density lipoproteins (LDLs) (Jürgens et al., 1995), whose oxidation is crucial in promoting atherogenesis in humans.

The depletion of plasmalogens, or their reduced amount, is associated with lipid raft microdomain (a cholesterol-rich membrane region) stability and raft involvement in cellular signaling (Pike et al., 2002; Munn et al., 2003; Rodemer et al., 2003). Both cholesterol and plasmalogen are of great significance in cell membrane fluidity and stability (Pike et al., 2002; Orgeig et al., 2003; Rodemer et al., 2003). Both may act as helper lipids to modify lung surfactant membrane structure (Lohner et al., 1991; Andersson et al., 2017). Model membrane system consisting of plasmalogens may go through the transformation from lamellar gel to liquid crystalline at the lower temperature (4–5°C) compared with their diacyl counterparts (Lohner, 1996). More striking is the observation that plasmalogens promote non-lamellar structures (including H_II_) at or below 30°C, while the diacyl analogs at much higher temperatures (Lohner, 1996; Nagan and Zoeller, 2001). These non-lamellar structures result in increased leakage of membranes and promotion of membrane fusion (Lohner, 1996; Nagan and Zoeller, 2001). This might be significant in certain cell processes such as endocytosis and exocytosis, highly depending on membrane fusion (Nagan and Zoeller, 2001). Plasmalogens are a key player in promoting H_II_ phase structure in biomembranes and might be important in membrane fusion-mediated events. This biophysical property of plasmalogens has been suggested to link to the potential role in facilitating cell membrane and intracellular molecule trafficking (Dean and Lodhi, 2018).

Of particular interest, human leukocytes (neutrophils) are also enriched in plasmalogens (Gottfried, 1967; Getz et al., 1968; Mueller et al., 1982, 1984; Sugiura et al., 1982; Tencé et al., 1985; MacDonald and Sprecher, 1989; Manning et al., 1995). Membrane plasmalogen level may determine the characteristics of plasma membrane, such as the formation of lipid rafts, which are crucial for efficient signal transduction and optimal phagocytosis of macrophages (Rubio et al., 2018). Plasmalogens may thus modulate the phagocytotic activity of macrophages (Rubio et al., 2018). Since both SP-A and SP-D are part of the innate immunity of cell and may thus modulate the phagocytotic activity of macrophages (Vaandrager and van Golde, 2000; Matalon and Wright, 2004; Pastva et al., 2007). In an *in vitro* study, the alveolar lining materials obtained from rats have been shown to enhance the bactericidal capacity of alveolar macrophages against *Staphylococcus aureus* (Juers et al., 1976). It deserves further studies on both lung surfactant proteins (SP-A, SP-D) and how they may work together with plasmalogens to modulate the function of macrophages through their membrane.

## Antioxidant Property of Lung Surfactant: Focus on Plasmalogens

Lung tissues of preterm newborns are particularly sensitive to the exposure of high oxygen as level compared with staying inside the womb of the mother. Moreover, the main treatment options for NRDS, oxygen supplementation and mechanical ventilation, are both known to promote oxidative stress and regional pro-inflammatory responses (Worthen et al., 1987; Pierce and Bancalari, 1995). Endotracheal surfactant therapy may prevent oxidative damage of the alveoli (Matalon et al., 1990). The bronchoalveolar lavage of preterm neonates treated with surfactant presented lower levels of pro-oxidant markers than untreated neonates (Dani and Poggi, 2014). It was demonstrated in the animal model that the natural calf lung surfactants contain a measurable amount of superoxide dismutase (SOD) and catalase (CAT), and both antioxidant enzymes have been demonstrated to exert consistent scavenging activity when incubated with a definite amount of H_2_O_2_ (Matalon et al., 1990). The administration of exogenous surfactant has been shown to decrease the oxidative damages in the lungs of mice (Machado et al., 2018). However, the main antioxidant activity of surfactant probably depends on different mechanisms, which can be enzymatic or non-enzymatic scavenger molecules naturally contained in surfactant mixtures (Cantin et al., 1990). Plasmalogens and polyunsaturated PLs are the main molecules putatively responsible for non-enzymatic antioxidant activity of natural lung surfactants (Yavin and Gatt, 1972; Khaselev and Murphy, 1999; Farooqui and Horrocks, 2001; Zemski Berry and Murphy, 2005; Richard et al., 2008).

Analysis of murine lung surfactant revealed several plasmalogen PE lipid species, encompassing ∼38% of total PE species. Upon exposure of ozone to murine surfactant, plasmalogen PE as sacrificing molecules preferentially reacted, as compared with their diacyl counterparts (Wynalda and Murphy, 2010). Interestingly, peroxisome numbers were substantially increased in Clara and alveolar type II cells, implying an increased requirement for peroxisome metabolism that may secure sufficient plasmalogen synthesis (Karnati and Baumgart-Vogt, 2009). Since the lung is a direct target of ROS, plasmalogens might protect against respiratory diseases in general by virtue of their role as an antioxidant. Finally, although not using a plasmalogen-deficient cell line, Zoeller et al. (2002) showed that increasing plasmalogen levels in human pulmonary artery endothelial cells protected them in response to oxidative stress by prolonging survival and reducing ROS accumulation.

Premature infants who received surfactant replacement preparations with higher amount of plasmalogens showed much better respiratory outcomes (Rüdiger et al., 2005). Interestingly, plasmalogens in type II alveolar cells are composed of 93% plasmalogen PE and 7% plasmalogen PC, while plasmalogens isolated from the lung surfactant contain 36.5% plasmalogen PC and 63.5% plasmalogen PE (Rüstow et al., 1994). This discrepancy may partially explain the membrane structure conversion of a lamellar body (LB) (in type II alveolar cell) to TM (at alveolar surface), and it deserves further exploration and studies.

## 3D Membrane Structures of Lung Surfactant

Structure and function are most likely interdependent and interrelated. As for lung surfactant, it undergoes three major structural transformations, namely, in the form of intracellular and extracellular LB, extracellular TM, and a surface monolayer (Sorokin, 1966; Weibel et al., 1966; Weibel and Gil, 1968; Kikkawa, 1970). Each of these forms has a distinct function, based on the structures they possess. First, lung surfactants containing LB are secreted by type II alveolar cells. Upon the release from type II alveolar cells, LB may convert to TM, which then forms a monolayer (Gil and Reiss, 1973; Paul et al., 1977). A lipid monolayer enriched in DPPC covering the air–water interface is considered to be responsible for the low surface tension. LBs are attached to the lipid monolayer, and together they make up the surface film, as visualized by transmission electron microscopy (TEM) (Veldhuizen and Haagsman, 2000). Though LBs have specific activity, which could directly adsorb and form surface-active films without being first converted into TM, the intact alveolar surfactant (a surface film that consists of a monolayer and a bilayer) performed better along with time (Magoon et al., 1983). The lung lavage subfractions rich in TM absorbed rapidly to air–liquid interfaces and reduced surface tension to very low values upon compression of the surface, and the preparations lacking TM were much less surface active, even though they contained identical PL composition (Thet et al., 1979; Young et al., 1992). Appearance of TM may give rise to a PL monolayer at alveolar air–liquid interface, and this PL monolayer together with LB may further elaborate the actual function and activity of lung surfactant (Weibel et al., 1966; Paul et al., 1977).

**FIGURE 3:**
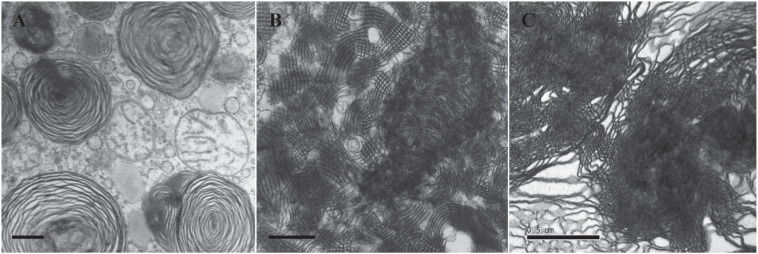
Transmission electron microscopy (TEM) micrographs of lung surfactant membrane structures and liposome construction of ameba-derived lipids. **(A)** Lung surfactant membrane structures display mainly as lamellar body (LB) in a premature infant with neonatal respiratory distress syndrome (NRDS). **(B)** Tubular myelin (TM)-rich surfactant membrane structure from a full-term newborn without NRDS. **(C)** Liposomal construct using cubic membrane-derived plasmalogen-rich ameba lipids. TEM images of **(A,B)** are adapted from deMello et al. (1987) with permission. All scale bars = 500 nm.

In order to compare the different membrane structures of lung surfactant from patients dying with/without NRDS, the lungs of 35 infants with NRDS and 19 without NRDS as control were examined by deMello et al. (1987) through light microscopy and electron microscopy. TEM data showed that the TM structure was not observed in the lungs of 35 infants who died with NRDS (Figure 3A), without exception. Abundant TM membrane structures, identified as characteristic lattice form (Figure 3B), were distinctly observed in 16 out of 19 control samples. TM as an intermediate phase between LB and monolayer appears indispensible in healthy lungs (Weibel et al., 1966; Gil and Reiss, 1973; deMello et al., 1987) and is thus considered as one of the polymorphic membrane structures of lung surfactant to act properly under normal physiological condition.

From the above-mentioned study, it is apparent that the absence of TM at alveolar surface of infants dying with NRDS showed a decrease in surface monolayer, which might finally lead to inactivity of lung surfactant. Therefore, it is important to know the key components that may contribute to the structure attribute of TM. Suzuki et al. (1989) have reconstituted TM *in vitro* with Ca^2+^ in a mixture of synthetic lipids (DPPC and PG) and surfactant proteins (SP-A and SP-B). The significance of Ca^2+^ on TM formation has been further evidenced by Sanders et al. (1980) and Benson et al. (1984).

TM was described by a tetragonally deformed P-based cubic surface (Figure 2B) (Schröder-Turk et al., 2006; Larsson and Larsson, 2014; Ninham et al., 2017), which is also used to depict intracellular cubic membranes (CMs) (Landh, 1995; Almsherqi et al., 2006, 2009). CMs represent highly curved, 3D nano-periodic membrane structures that correspond to mathematically well-defined triply periodic minimal and level surfaces (Landh, 1995; Almsherqi et al., 2006, 2009; Deng et al., 2017). Experimental data from ameba Chaos studies showed that CM is enriched with unique ether PLs, plasmalogens carrying very long-chain polyunsaturated fatty acids (PUFAs) (Deng et al., 2009, 2017; Deng and Almsherqi, 2015). Deng and Almsherqi (2015) proposed that these PLs not only facilitated CM formation but also together might act as an antioxidant defense system to provide a protective shelter for RNA (Deng and Almsherqi, 2015). The potential interaction of CM with RNA may reduce the amount of RNA oxidation and promote more efficient protein translation (Deng and Almsherqi, 2015; Deng et al., 2017). Specifically, intracellular membranes may transform into CM organizations in response to multiple pathological, inflammatory, and oxidative stress conditions (Almsherqi et al., 2009; Deng and Almsherqi, 2015). CM has been observed in numerous cell types from all kingdoms and in virtually any membrane-bound subcellular organelles (Almsherqi et al., 2009).

## Plasmalogens May Modify Phase Structures of Phospholipid Mixtures

Perkins et al. (1996) demonstrated that a lipid mixture with dioleoylphosphatidylethanolamine (DOPE):DPPC:cholesterol in a molar ratio of 7:3:7 at 37°C appeared to be a co-existence structure of lamellar and H_II_ phases. The H_II_ transition might facilitate lipid transfer to air–water interface through unstable transition intermediates (Possmayer et al., 1984). The spreading rate of this lipid mixture formulation is similar to that observed in Curosurf^®^ natural surfactant preparations (Perkins et al., 1996), suggesting that lipid polymorphic phase behavior might be essential to maintain surfactant activity and function. It is interesting to note that Curosurf^®^ is a porcine surfactant with the highest content of plasmalogen and higher performance and efficiency than other commercially available nature surfactant products (Rüdiger et al., 2005).

It is important to establish the potential role of plasmalogens in 3D membrane structure of lung surfactant. Lohner et al. (1991) first reported the findings on biophysical property of plasmalogens in promotion and stabilization of non-lamellar membrane phase structure. The lipid mixtures of PE and PC of natural and synthetic surfactant preparations tend to assume the LB-like structure. The curved H_II_ phase structure is unlikely to be seen when the content of PC in lipid mixture is high (>25 mol%) (Cullis and de Kruijff, 1978; Cullis et al., 1978), suggesting that the role of PC is to stabilize the planar bilayer structure of biomembrane. Nevertheless, this circumstance can be modified and reversed by plasmalogen, a curvature-modified ether lipid (Lohner et al., 1991). In the presence of 38 mol% PC and 62 mol% plasmalogen, H_II_ phase is formed at 60°C (Lohner et al., 1991). The biophysical property of plasmalogens may determine the optimized packing of H_II_ at interface area, and this could be well-explained by their unique conformation of vinyl ether bond at sn-1 chain (Cullis et al., 1978; Malthaner et al., 1987). Nevertheless, the role of plasmalogens as structural attributes in lung surfactant requires more studies to be confirmed.

## Plasmalogens May Modify Surface Tension and Surface Viscosity of Surfactant-Like Phospholipid Mixture

The optimized performance of surfactant-like PL mixture with plasmalogen modification was first reported by Rüdiger et al. (1998). The measurements of surface tension in lipid mixtures with different concentrations of plasmalogens were analyzed and compared. The experimental data revealed that plasmalogens effectively lowered the surface tension of surfactant-like PL mixtures (Rüdiger et al., 1998). Only 2% plasmalogen of total PLs was able to dramatically lower the surface tension of about 10 mN/m (Rüdiger et al., 1998), suggesting that plasmalogen-modified lung surfactant preparations could effectively reduce the surface tension and might further promote the mechanics of breathing.

Highly PUFA-containing PLs (PUFA-PLs) and plasmalogen content in tracheal aspirate of preterm infants may reduce the risk of developing chronic lung diseases (Rüdiger et al., 2000). So far, biophysical properties of lung surfactant are best described and characterized with two parameters, surface tension and surface viscosity (Rüdiger et al., 2005). In order to clarify the relations between lipid mixtures and surface viscosity of lung surfactant, the lipid composition and surface properties of three commercial surfactant preparations (Alveofact^®^, Curosurf^®^, and Survanta^®^) were examined and compared (Rüdiger et al., 2005). The experimental data suggested that either vinyl ether link plasmalogen or ester link PUFA-PLs are both required for lowering the surface viscosity of the surfactant mixtures (Rüdiger et al., 2005); however, plasmalogen is superior and more effective than PUFA-PLs (Tölle et al., 1999). With the deeper understanding and appreciation of this special ether lipid molecule, it becomes clear that plasmalogens might indeed play a vital role in activity and function of pulmonary surfactant.

## Conclusion

Plasmalogens are not only a structural component of biomembranes and a reservoir for secondary messengers, but they also participate in multiple cell processes, including membrane fusion, cholesterol efflux, and antioxidation (Nagan and Zoeller, 2001; Lessig and Fuchs, 2009; Wallner and Schmitz, 2011; Luoma et al., 2015). The membrane plasmalogen level may determine characteristics of cell membrane such as membrane fluidity and formation of lipid rafts microdomains, which are essential for efficient signal transduction prepared for optimal phagocytosis of macrophages (Rubio et al., 2018).

Two proposed roles of plasmalogens may serve for proper activity and function of lung surfactant. First, plasmalogens are considered as powerful endogenous antioxidants that can be supported by experiment data directly (Wynalda and Murphy, 2010). The main function of lung is engaged in the air breathing, and plasmalogens may protect lungs from the damage of oxygen-free radicals potentially from air breathing. Second, plasmalogens may promote non-lamellar (cubic or hexagonal) phase formation *in vitro* (Lohner, 1996; Koivuniemi, 2017). The presence of non-lamellar phase may speed the spreading rate of lipid mixture formulation, and the spreading rate is similar to that observed in Curosurf^®^ (Perkins et al., 1996). There is still lack of direct evidence to support that plasmalogens may promote non-lamellar membrane structure such as TM in lung surfactant; nevertheless, the *in vitro* lipidic phase studies (Lohner et al., 1991; Lohner, 1996) in addition to the liposomal construction data (Figure 3C) by using ameba Chaos CM-derived lipids rich in plasmalogens (Deng et al., 2009) have led to the current hypothesis of having potential contribution of plasmalogens in non-lamellar TM (CM-like) formation of lung surfactant. Of great interest, an extensive literature survey has revealed that intracellular membranes may rearrange into CM organization in response to external and/or internal environmental cues (Almsherqi et al., 2009), especially oxidative stress (Deng and Almsherqi, 2015; Deng et al., 2017), and the evolution of CM has been proposed as an antioxidant defense system in the eukaryotes (Deng and Almsherqi, 2015).

**FIGURE 4:**
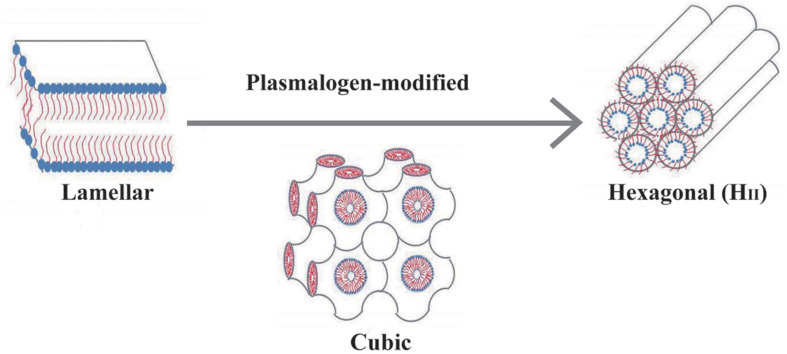
A schematic drawing demonstrates that plasmalogen modification may promote lamellar to non-lamellar (cubic and hexagonal) phase transitions. Lipidic phase structures were adapted from Huang and Gui (2018) with permission.

A schematic representation in Figure 4 demonstrates how plasmalogen modification may be involved in membrane phase transitions in general. In this report, we simply and humbly intend to introduce the potential roles of plasmalogens in lung surfactant, as they may provide vital functions as antioxidation, phase modification, surface tension, and viscosity reduction in addition to immunity modulation even though they might be just a minor component of PLs in lung surfactant. A question emerges that whether a novel surfactant preparation without the requirement of surfactant proteins may provide a protein-free solution for the current synthetic surfactant products to avoid or eliminate the unwanted cellular immune responses potentially caused by surfactant proteins of animal origins. It might offer a novel synthetic surfactant preparations free from cultural and religion concerns, as compared with the preparations from the bovine or porcine origin.

## Author Contributions

YD and RZ conceived the review and wrote the manuscript. PR, JW, and RP revised the literature and helped to writing the manuscript. YD supervised the overall project and edited the manuscript. All authors had the opportunity to discuss and comment on the manuscript.

## Conflict of Interest

The authors declare that the research was conducted in the absence of any commercial or financial relationships that could be construed as a potential conflict of interest.
